# Surgical Management in Primary Congenital Glaucoma: Four Debates

**DOI:** 10.1155/2013/612708

**Published:** 2013-05-22

**Authors:** Ta C. Chang, Kara M. Cavuoto

**Affiliations:** Department of Ophthalmology, Bascom Palmer Eye Institute, University of Miami Miller School of Medicine, Miami, FL 33136, USA

## Abstract

Primary congenital glaucoma is a worldwide diagnostic and therapeutic challenge. Although medical management is often a temporizing measure, early surgical intervention is the definitive treatment. As the abundance of surgical treatment options continues to expand, the authors will compare and contrast the available options and attempt to provide a consensus on surgical management.

## 1. Background and Outcome of Primary Congenital Glaucoma

Ophthalmologists throughout the world are faced with the diagnostic and therapeutic challenge of primary congenital glaucoma (PCG). Often, infants present with the classic triad of blepharospasm, epiphora, and photophobia. Characteristically, the patients are male (65%) with bilateral involvement (70%) and diagnosed within the first year of life (80%) [1]. The natural course of congenital glaucoma results in pan-ocular dysfunctions, with vision loss resulting from Descemet breaks, corneal edema, and optic neuropathy, and eventually buphthalmos, and amblyopia. Prior to the advent of surgical and medical treatments, most of these children develop little or no useful vision in the affected eyes. 

Primary congenital glaucoma accounts for 0.01%–0.04% of total blindness [1]. The incidence varies with the “founder effect” and when high rates of consanguinity are present. PCG is typically autosomal recessive, ranging in incidence from 1 : 1250 in consanguineous Slovakian Roms [1] to 1 : 30,000 in Western countries [2]. The differential diagnoses for PCG is broad and would include congenital glaucoma associated with nonacquired ocular anomalies or systemic disease/syndromes. Hence, careful surveillance of any systemic comorbidity is crucial in the well-being of the patient, as some systemic syndromes may carry life-threatening abnormalities that require prompt attention (e.g., cardiac outflow tract abnormalities in Axenfeld-Rieger syndrome) [3]. Other types of childhood glaucoma, such as traumatic, uveitic, infectious, and steroid-induced should be ruled out with careful history and examination.

Appropriate and timely diagnosis and treatment can dramatically alter the course of disease and restore visual development. In assessing the risk/benefit profile of medical versus surgical treatment, angle surgery offers a high chance of success with low complication rates. Hence, surgery is often considered first-line therapy. However, medical therapy provides a temporizing measure to clear the cornea to facilitate examination and surgical intervention. Alpha-agonists are contraindicated in infants due to high risk of central depression [4]. Topical beta-blockers have been studied extensively and show improvement in intraocular pressure in approximately one-third of patients in multiple studies [5–7]. Cautious use of this class of medication is advised as beta-blockers may lead to respiratory complications in predisposed young children. For this reason, selective beta-blocker (e.g., betaxolol) is preferred over nonselective ones, and the lower concentrations than typical adult formulations are used. The ophthalmologist should communicate with the pediatrician to assure there are no cardiac or pulmonary contraindications. Carbonic anhydrase inhibitors are generally safe and well tolerated. To avoid severe metabolic acidosis, topical drops are preferred to systemic suspensions. Prostaglandin analogues have been tried however showing mostly nonresponders in the PCG group [8].

## 2. Surgical Approach

Historically, Hippocrates described a child with likely congenital glaucoma as early as the 5th century BCE. Nearly two thousand years later, PCG retained a poor prognosis despite recognition of the condition and various attempts at surgical control. It is thought that goniotomy was first described in 1893 by Carlo de Vincentiis; however it was performed without visualization of the angle and resulted in poor outcomes [9]. It was not until 1938 when Otto Barkan performed the first goniotomy utilizing gonioscopy and a knife to incise the trabecular tissue [1]. Although goniotomy was difficult or impossible in eyes with significant corneal edema, it became the standard of treatment for PCG. Trabeculotomy, which could be performed in the presence of corneal opacity, was later developed by Smith in 1960 [10]. Both of these procedures have excellent success rates and continue to remain part of every pediatric glaucoma surgeon's arsenal of techniques to manage intraocular pressure in PCG. 

*Debate 1: Reinvestigating the Basics: Goniotomy versus Trabeculotomy Which Is Better?*. Goniotomy is designed to create a route for aqueous drainage through Schlemm's canal by incision of the trabecular meshwork under direct visualization. Pilocarpine is often used to constrict the pupil and a goniolens is used for visualization of angle structures. The trabecular meshwork is incised with a goniotomy knife or small gauge needle for 4-5 clock hours with a single incision. To perform the procedure, an adequate view of the angle and knowledge of angle anatomy are required. If corneal edema is present, it is sometimes possible to treat it medically for a short time to reduce edema. It is also possible to remove the corneal epithelium to improve visibility. The success of goniotomy is thought to be approximately 80% with a single procedure, especially if recognized and treated within the first year of life [1]. Benefits of goniotomy are that it is minimally traumatic to the surrounding tissues, does not result in conjunctival scarring, and has a shorter operating time. However, multiple incisions are to treat the entire angle. Complications of goniotomy include hyphema, iridodialysis, peripheral anterior synechiae, cyclodialysis, and lenticular injury.

Trabeculotomy involves disrupting the tissue between Schlemm's canal and the anterior chamber using an ab externo approach to create direct communication. Currently, there are at least three different approaches: rigid probe, suture, and illuminated microcatheter. Pilocarpine is used for pupillary constriction and a peritomy is typically performed. A partial-thickness scleral flap is created and Schlemm's canal is identified, incised, and cannulated for 360 degrees. High success rates ranging from 87% to 92% in cases of PCG presenting before one year of age make trabeculotomy an excellent procedure [9]. Other benefits of trabeculotomy are that it can be performed with a cloudy cornea and one incision can be used to treat the entire 360 degrees. Like goniotomy, a detailed understanding of angle anatomy is required to perform the procedure. Complications include hyphema, unintentional filtering blebs, choroidal detachment or hemorrhage, and false passages into the eye. Compared to goniotomy, trabeculotomy takes more time and causes conjunctival scarring which may compromise the future options of placing glaucoma drainage implants or perform a trabeculectomy. However, if the procedure is done temporally it does not compromise subsequent filtration or implant surgery. 

Due to the history of success with both procedures with well-defined patient parameters, this is the most straightforward of the “debates.” Goniotomy is preferred in children less than one-year old with good visibility of angle structures. Trabeculotomy is the procedure of choice in children with poor visualization of angle structures [9, 12], although some surgeons advocate filament-assisted circumferential trabeculotomy over goniotomy as a primary procedure even in PCG patients with clear corneas due to greater success when compared to goniotomy [13].

**Figure 1:**
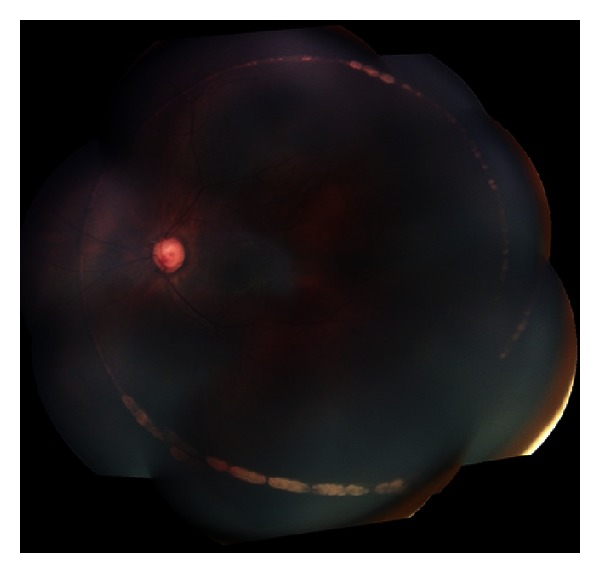
Color fundus photography demonstrating circumferential chorioretinal scarring after misdirection of blunted 6-0 polypropylene suture used in filament-assisted circumferential trabeculotomy (photograph courtesy of Ms. Ditte Hess).

*Debate 2: The Evolution of the Basics: Is Rigid Probe Trabeculotomy Superior to Filament-Assisted Trabeculotomy?* Smith's initial description of trabeculotomy ab externo in 1960 involves passing a nylon filament around Schlemm's canal via an external radial incision and rupturing the trabecular tissue in a drawstring fashion [10]. This approach has the advantage of incising all 360 degrees of angle, thus avoiding multiple treatments. However, potential for false passages and the inability to visualize or control the filament's position can be problematic. Smith's follow-up publication in 1962 described a complication in which the distal end of nylon filament was not recovered from the opposing Schlemm's canal cut-down opening [14]. Filament entering the anterior chamber, suprachoroidal or subretinal spaces can cause serious complications, including damage to the cornea and lens, hyphema, and chorioretinal scarring (Figure 1).

**Figure 2:**
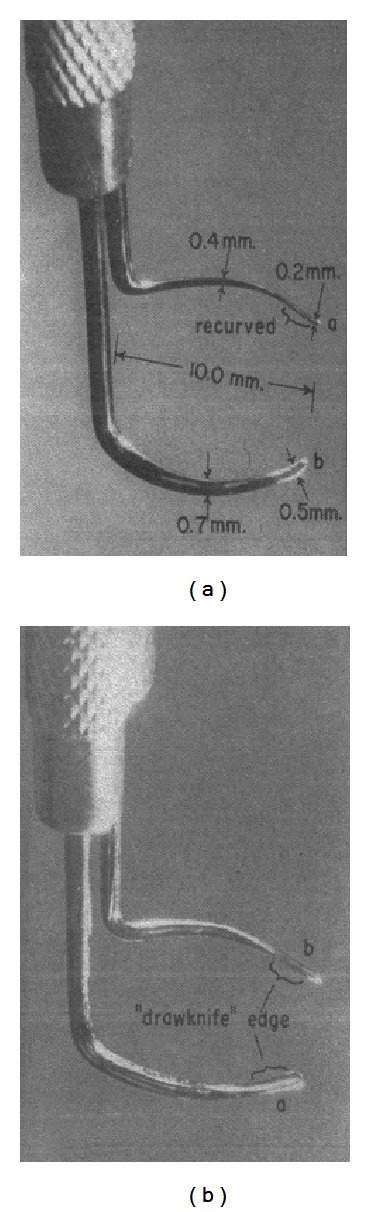
Two varieties of rigid probes as described by Allen and Burian and used in sector trabeculotomy. The probe on the left has a blunt tip, while the one on the right is fitted with a drawknife edge [11].

**Figure 3:**
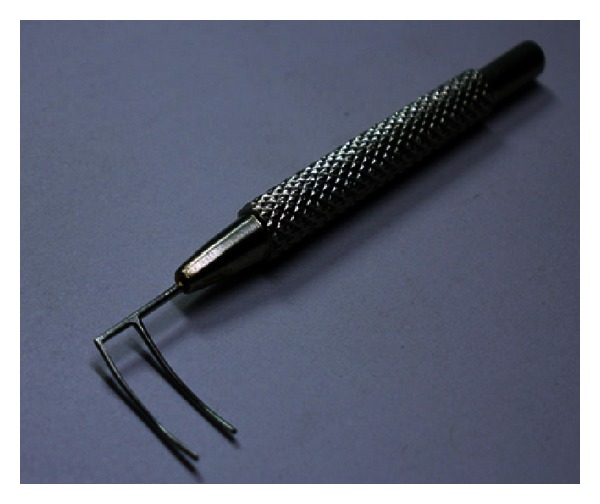
Harms trabeculotomy probe with double parallel prong design.

Allen and Burian described an alternative technique of trabeculotomy using specifically designed rigid probes (Figure 2) that achieved sector opening of the trabecular tissue in 1962 [11]. Compared to the flexible nylon filament or the later-adapted polypropylene filament as described by Beck and Lynch [15], the rigid probe offers better directional control once it enters the Schlemm's canal, and the later double parallel-pronged design (as in the Harms trabeculotomy probes, Figure 3) helps the surgeon determine the probe's position in the canal and anterior chamber. A single incision allows opening of approximately 120 degrees of angle. In the case of treatment failure, subsequent angle surgery is usually offered prior to considering glaucoma drainage device implantation. 

There are no well-designed, large prospective double-masked controlled trials comparing the outcomes of trabeculotomy performed with a filament to the outcomes performed with a rigid probe, although several smaller retrospective reports lend insight into the efficacy of each technique. The definition of “success” varies widely between studies, although most criteria indicated clinically stable result. Using the filament technique, Beck and Lynch reported 15 PCG patients (mean age 8.5 months), 87% of whom achieved treatment success (IOP ≤ 22 mm Hg without medication, stabilization or improvement of disc appearance, absence of progressive corneal enlargement, or axial length increase) after mean followup of 12 months [15]. Mendicino et al. in comparing filament-assisted trabeculotomy with goniotomy reports on 24 eyes in PCG patients (mean age 4.5 months) that had undergone circumferential trabeculotomy with a filament, 92% of which with favorable treatment outcome (IOP less than 22 mm Hg with or without medications, no progressive disc changes, no additional surgeries) after mean followup of four years [13]. More recently, Sarkisian reports a series of 16 eyes in which filament trabeculotomy was successfully performed using an illuminated microcatheter in children under 3 years of age diagnosed with PCG. Circumferential canulation was achieved in 75% of these eyes, and the cohort's mean intraocular pressure averaged under 21 mm Hg after 6 months followup although criteria for treatment success was not defined nor was the population of sufficient size to allow statistical analysis. The illuminated microcatheter allows continued visualized of the catheter tip during canulation, hence reducing the risk of undetected catheter misdirection [16].

Dietlein et al. [17] compared rigid-probe trabeculotomy to combined trabeculotomy/trabeculectomy and to trabeculectomy alone in children. In their trabeculotomy arm (17 of the 61 trial patients), they reported treatment success (IOP < 18 mm Hg under general anesthesia or <21 mm Hg while awake, stable axial length and optic nerve appearance, clear cornea) of 77% at 6 months and 59% at 2 years. Treatment success extrapolated from the published life table at 1 year is approximately 60%. In a consecutive series of 46 patients followed for an average of 38 months, Filouš and Brunová [18] reports a cumulative success of 87% (success defined by IOP less than 21 without progressive corneal or optic disc changes), 22% of whom required additional angle surgery after the initial rigid-probe trabeculotomy. 

In summary, rigid-probe trabeculotomy has an overall success of approximately 60%–87% after a mean followup of 1–3 years, with a subset of the patients requiring additional angle surgery. A 360° filament trabeculotomy, on the other hand, achieved surgical success in 87%–92% of patients after 1–4 years of followup. No significant complications were noted in any of the trials. The illuminated microcatheter improves the safety of filament trabeculotomy by allowing continuous visualization of filament tip and allows rapid detection of misdirection. Given the available outcome data and the improved safety profile of illuminated microcatheter, the authors recommend filament trabeculotomy with an illuminated microcatheter over rigid-probe trabeculotomy as an initial procedure in PGC. 

*Debate 3: Trabeculectomy versus Tube Shunt in the Pediatric Congenital Glaucoma Group: Are the Results the Same?* After failing treatment of 360 degrees of angle (whether one-time filament trabeculotomy or multiple-session with goniotomy/rigid-probe trabeculotomy) and maximizing medical treatment, the next procedure of choice often depends on surgeon's training and practice style. Most surgeons favor either a shunting procedure, that is, a glaucoma drainage device (GDD), or a filtering procedure, usually a trabeculectomy (with or without augmentation with antifibrotic agents). The two choices have different risk/benefit profiles. Trabeculectomy does not involve a foreign-body implant and the associated complications including corneal trauma, motility disturbances, and exposure. However, the exuberant cicatricial response in these patients often results in low success in intraocular pressure control. Antifibrotic agents may improve IOP control, but their use increases the chance of serious bleb-related complications. On the other hand, glaucoma drainage devices may offer greater IOP control over long-term followup, but the risk of implant-related complications may increase over time. Some argue that initial filtering procedures might “keep more options open” as there is an inherent bias toward implanting a second GDD after the first one fails instead of performing a trabeculectomy, while a failed trabeculectomy does not preclude subsequent GDD procedures. 

In the evaluation of trabeculectomy versus glaucoma drainage device implantation after failure of angle surgery, we must consider both long-term outcome and the risk of complications. There is a paucity of clinical data regarding the efficacy and safety of trabeculectomy versus glaucoma drainage devices in PCG. Most retrospective reviews on this subject included patients of various subtypes of childhood glaucoma or combined surgical techniques. Some of these studies did perform data analysis based on glaucoma subtypes, which lends insight into our clinical debate. 

Initial studies argued against the consideration of trabeculectomy in the treatment of PCG. In 1979, Beauchamp and Parks reported 50% success in children who had undergone trabeculectomy after 10–39 months of followup, with numerous complications including vitreous loss, retinal detachment, and endophthalmitis [19]. Subsequent studies suggested that there was some utility in performing trabeculectomies in children with PCG. Fulcher et al. [20] retrospectively reviewed 13 eyes with PCG that had undergone trabeculectomy without MMC with 5–14 years of followup and reported an overall success of 92.3% after 1 single trabeculectomy and 100% success with two trabeculectomies at the last follow-up visit. The authors reported no serious complications in any patients. 

Due to the exuberant fibrotic response to surgery in children, several authors have investigated the use of antifibrotic agents to increase the chances of surgical success. Al-Hazmi et al. [21] reviewed 254 eyes (including 98% PCG) that had undergone trabeculectomy augmented with mitomycin-C (MMC). They report age-related success rates of between 32% (age less than 6 months at time of surgery) to 85% (aged 49–85 months of age) after at least one year of follow-up. However, the authors also reported age-related rates of complications between 0% (age less than 6 months) and 50% (age 49–84 months) after at least 1 year of follow up. The most frequent complication was the development of cystic bleb, followed by hypotony and bleb leak. Sidoti et al. [22] reviewed a cohort of 15 eyes with PCG which had undergone trabeculectomy with MMC and reported treatment success of 87% of patients at 12 months, 78% at 24 months, with a cumulative bleb-related infection in 27% of this cohort at the last follow-up visit. Rodrigues et al. [23] retrospectively reviewed 91 eyes with PCG that had undergone trabeculectomy (61 without MMC and 30 with MMC) and found that, with a minimum followup of 2 years, long-term surgical outcome did not differ between the MMC versus non-MMC groups, though the MMC group had higher incidents of complication. Agarwal et al. [24] compared the effects of 0.2 mg/mL and 0.4 mg/mL MMC in trabeculectomy and included 17 patients with PCG. They noted no difference in success rate between the two concentrations, although the 0.4 mg/mL group had a higher complication rate (33.3% in 0.2 mg/mL group versus 66.6% in 0.4 mg/mL group over 18 months). The overall success rate at 18 months was approximately 60%–86.7%. Overall, the success rate of trabeculectomy is difficult to interpret based on the data presented, but from the life table extrapolation, approximately 50% of the surgery fail within the first 5–10 years.

Netland and Walton [25] first introduced glaucoma drainage devices to be used in pediatric glaucoma. Since that time, various GDDs have been utilized for intraocular pressure control in these children, including Baerveldt, Molteno, and Ahmed devices. In a retrospective case-control study, Beck et al. [26] identified 46 pediatric eyes with various glaucoma subtypes that had received aqueous shunt device prior to age of 24 months and compared the outcome to age-matched eyes that received MMC-augmented trabeculectomy. At the last followup, 20.8% were considered successful in the trabeculectomy group (mean followup 11.5 months) while 71.7% were considered successful in the aqueous shunt devices group (mean followup 31.5 months). The trabeculectomy group had an 8.3% cumulative incidence of endophthalmitis, while 45.7% of aqueous shunt device group required more surgical procedures, such as tube repositioning, due to complications of tube implantation.

The efficacy and complication rates of trabeculectomy with MMC and the valved Ahmed glaucoma implant augmented with MMC were compared in a randomized clinical trial by Pakravan et al. [27] in a study involving 30 aphakic eyes with glaucoma. Although the study focused on aphakic eyes with glaucoma, an entity quite distinct from PCG in pathophysiology, onset, presentation, and outcome, the authors' prospective randomized method allows a head-to-head comparison between the two surgical approaches, which lend insight into the efficacy and safety of each. The authors' surgical technique in tube implantation does not include concurrent pars plana vitrectomy to minimize the risk of vitreous prolapse and tube obstruction. The authors defined success as IOP between 5 and 21 mm Hg. If an eye did not require medication for IOP control, it was called a “complete” success, and if medication was needed it was designated a “qualified” success. With 15 eyes in each arm, the trabeculectomy group, after a mean followup of 14.8 months, had an overall success of 73.3% (including 33.3% complete and 40% qualified success), while the Ahmed group, after mean followup of 13.1 months, enjoyed an overall success of 86.7% (20% complete and 66.7% qualified success). There were no significant differences between the visual acuity outcomes between the two groups, while there were 40% complications in the trabeculectomy group and 26.7% in the Ahmed group. 

In the comprehensive review by Ishida et al. [28], the authors collimated the outcomes of twenty-one studies of glaucoma drainage implants in pediatric patients between 1984 and 2004. The overall success ranged from 54% to 95% (mean approximately 75%), with success criteria generally based on IOP less than 22 mmHg (with or without medical) and after variable follow up lengths ranging from 12 to 124 months. The long-term effectiveness of intraocular pressure control has also been noted by several other groups; however the effectiveness must be balanced with the complications related to hypotony and to the tube itself [29–31].

In summary, after various durations of followup, trabeculectomy has a success rate at one year ranging between 32% and 100% with most studies reporting 50%–87%. The frequency of complications vary, although at the high end it is approximately 66.6% when MMC was utilized in conjunction with the surgical procedure. Surgical efficacy decreases over time, young patients (specifically those under 6 months of age) tend to do poorly, and augmentation with MMC makes little or no differences in success rate but may increase the risk of complication. Tube shunt, on the other hand, has an overall success of approximately 75%–86.7% after 1-2 years and a rate of severe complication of between 26.7% and 45.7% in the same time period. With comparable efficacy and a lower complication rate, tube shunt surgery seems to be the favored procedure in children with PCG who have failed angle surgery.

*Debate 4: On the Horizon: Looking at the Alternatives in Pediatric Congenital Glaucoma Surgery.* Some surgeons advocate combined procedures or nonpenetrating surgeries as alternatives to traditional angle surgeries as primary procedures. Mullaney et al. [32] retrospectively reviewed 100 consecutive eyes with congenital glaucoma, 63% of which were PCG. These patients underwent combined rigid-probe trabeculotomy and trabeculectomy augmented with MMC. The authors reported success in 67% of patients after average followup of 10 months (success defined as IOP < 21 mm Hg). Within the PCG subgroup, the 78% of patients had surgical success over 10 months, which compares well with other series with rigid-probe trabeculotomy alone. This suggests that the addition of trabeculectomy with MMC to the rigid-probe trabeculotomy offers no additional advantage and presumably would increase the risk of bleb-associated complications. 

**Figure 4:**
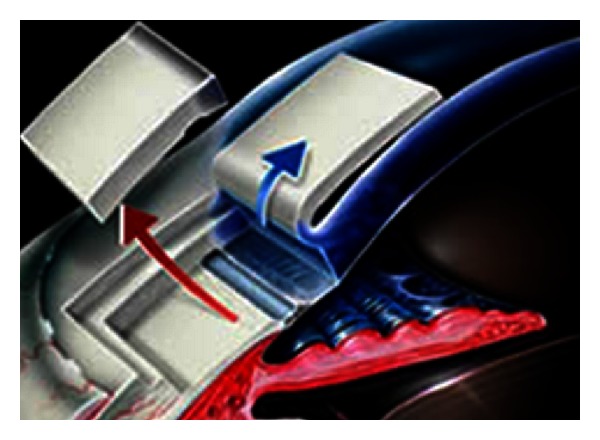
Schematic drawing demonstrating techniques of deep sclerectomy.

Nonpenetrating procedures are of particular interest in pediatric patients because they are assumed to have a decreased risk of hypotony, infection, lens injury, Descemet detachment, and other intraocular trauma. Deep sclerectomy involves the unroofing of Schlemm's canal under a sclera flap, with concurrent removal of the juxtacanalicular trabecular tissues without entering into the eye (Figure 4). The procedure leaves behind a trabeculodescemetic window that provides the resistance to aqueous drainage to prevent hypotony. Al-Obeidan et al. [33] prospectively followed 143 eyes of 120 patients with congenital glaucoma without concurrent anterior segment anomalies (90.9% diagnosed with PCG). Non-penetrating deep sclerectomy was technically successfully in 74 eyes (52.4%), with the remainder converted to penetrating procedures with either involuntary perforation of the trabeculodescemetic window or an intentional perforation due to inadequate aqueous percolation. Within the group in which non-penetrating deep sclerectomy was performed, success (IOP under 21 mm Hg) after mean followup of 35.8 months was 82.4% (79.9% complete success, 2.7% qualified success), while the cohort with penetrated deep sclerectomy had complete and overall success in 84.1% and 89.9% of patients, respectively. The surgical technique of deep sclerectomy proved to be difficult, as in the authors' experienced hands only 51.7% of eyes had successful completion for the non-penetrating procedure. Furthermore, the authors' data seem to suggest that perforation does not seem to worsen outcome or increase complication rates, which challenges the notion that non-penetrating procedures offer greater safety profile in this patient population. Intraoperative use of mitomycin-C and nonabsorbable space-maintaining material may negate the benefits of this minimally traumatic approach by increasing the future (and life-long) risk of hardware exposure and scleral ectasia. 

Viscocanalostomy is a non-penetrating procedure first described by Stegmann et al. [34]. This procedure combines both deep sclerectomy with unroofing of Schlemm's canal and dilation of the Schlemm's canal with injection of sodium hyaluronate into the canal to provide a physical barrier to fibrinogen migration. The results show promising IOP reduction in adult patients who had previously been uncontrolled on medical therapy, with a postoperative IOP of 22 mm Hg or less without medical therapy in 82.7% of eyes with an average followup of 35 months. Noureddin et al. [35] conducted a pilot study of eight consecutive infants diagnosed with bilateral PCG, with the more severe eye undergoing randomization to either trabeculotomy or to viscocanalostomy. The observation was similarly well-controlled mean IOP between the two groups with a followup of twelve months. Kay et al. [36] reported a series including 19 surgically naïve PCG eyes that underwent modified viscocanalostomy. In addition to classically described viscocanalostomy, the author performed stab incisions that entered the anterior chamber adjacent to the ostia, hence converting this procedure into a penetrating procedure. After mean followup of 20 months, the overall success was 89% without serious intraoperative or postoperative complications noted. 

In summary, based on the literature reviewed, the addition of trabeculectomy to trabeculotomy does not seem to offer significant advantage in success rate and may increase the risk of complications. Non-penetrating deep sclerectomy may hold promise given its theoretical benefits over penetrating surgeries but may be technically difficult to perform in inexperienced hands. Viscocanalostomy may offer an alternative to classic angle surgery if it is demonstrated to be safe and noninferior.

## 3. Summaries

1. Goniotomy and rigid-probe trabeculotomy are both successful in treating PCG. Goniotomy has a shorter operating time and does not cause conjunctival scarring but is limited to cases with a clear cornea. While goniotomy is traditionally offered as primary procedure in PCG patients with clear cornea, the improved success rate and safety profile of circumferential trabeculotomy may offer greater advantage over goniotomy.
2. Filament-assisted trabeculotomy has superior success rates in the literature when compared to rigid probe trabeculotomy. Illuminated microcatheters allow improved visualization of the catheter tip during canulation, reducing the risk of misdirection. Filament trabeculotomy with an illuminated microcatheter is superior to rigid-probe trabeculotomy as an initial procedure.
3. After angle surgery, trabeculectomy may be useful in children to avoid the implantation of a glaucoma drainage device; however approximately 50% fail in the initial 5–10 years. Augmentation of trabeculectomy with mitomycin-C does not appear to improve surgical outcomes however does increase complication rates, especially in high concentrations. The addition of trabeculectomy with MMC to the rigid-probe trabeculotomy seems to offer no additional advantage and presumably would increase the risk of bleb-associated complications. Glaucoma drainage devices seem to have a higher success rate and a lower complication rate when compared with trabeculectomy.
4. New techniques, including deep sclerectomy and viscocanalostomy, appear to offer excellent success rates while minimizing the risk of glaucoma surgery. Non-penetrating surgery offers both the efficacy of lowering IOP and guards against hypotony but is technically difficult to achieve. Viscocanalostomy may offer similar efficacy to traditional trabeculotomy, although more studies are needed for conclusive evidence.
